# The treatment of the reno-ureteral calculi by extracorporeal shockwave lithotripsy (ESWL)

**Published:** 2012-06-18

**Authors:** E Ceban

**Affiliations:** Surgical and Nephrology Department, “Nicolae Testemiţanu” University of Medicine and Pharmacy, Chisinau, Republic of Moldova

## Abstract

Urolithiasis has an important role in the structure of urological pathology, due to its high incidence, frequency of recurrence and complications it might cause. There are many methods of treatment for kidney stones described in the scientific literature as conservative, surgical, laparoscopic, endoscopic, and ESWL. In this study, we have analyzed the ESWL method of treatment of reno-ureteral stones.

There are still many controversies about the effectiveness of different models of lithotripters but the lithotripter type Modulith SLK Storz Medical (Germany) used in our clinic has proved to be very effective. ESWL is currently the first-line treatment for the majority of kidney and ureteral stones, which are up to 20 mm in diameter.

## Introduction

Urolithiasis has an important role in the structure of the urological pathology, due to its high incidence, frequency of recurrence and complications it may cause. It reduces the medium life span from 5 to 20% of the patients, the recurrences being detected in 50-67% of the cases [**18,25**].

Presently, in Republic of Moldova this illness is situated on the first place in the structure of the clinical urological illnesses [**6**]. The fact that the urolithiasis preponderantly affects the persons with a productive age, being very rare in elders and children, having a frequency of over 70% in the patients aged 20-25 years, which leads to the loss of the working ability [**13**]. According to the data of some authors [**7,29**] 8,9% of the men and din 3,2% of the women endure urolithiasis during their lifetime. The frequency of the pathology, the clinical particularities, the possible complications that might occur, the difficulties which appear in the process of the diagnosis and the treatment, highlight the necessity of studying the problems regarding the urolithiasis [**23,31**]. Many specialty methods of treatment of the urolithiasis are described, among which the following should be mentioned: surgical, conservative, endoscopic, laparoscopic, robotic and the extracorporeal lithotripsy with shock waves (ESWL). In the last years, the treatment of the urolithiasis has been permanently perfected thanks to the practical implementation of some different new methods such as: the extracorporeal shock wave lithotripsy, high and low endourology, percutaneous nephrolithotomy (NLP), transperitoneal and retroperitoneal laparoscopic pielolitotomy, robotic pielolitotomy [**4**]. Since 1991, ESWL has been also implemented in the Republic of Moldova. This method has radically changed the conceptions and the strategy of the specialists regarding the management of the treatment of urolithiasis [**6,20,22**]. According to the data of some authors it is the first most important one in the set of alternative methods of the reno-ureteral calculi treatment [**15,16**] with an efficiency of 80-98% [**31**]. The paper analyzes the extracorporeal lithotripsy method of treatment of the reno-urethral calculi in various aspects, according to the comparison, complications and results obtained from the two calculi disintegration devices. ESWL – (Extracorporeal Shock Wave Lithotripsy) is a noninvasive method of treatment of the urinary calculi by generating the shock waves outside the human organism and their focusing on the calculus [**1,2**]. The last ones are disintegrated in little fragments that can be spontaneously eliminated. The use of the sonic waves to fragment the urinary calculi is a process known by the urologists since 1950, the method being used by direct contact (for ultrasonic and electrohydraulic lithotripsy). The shock waves are less attenuated by the propagation through liquid or tissues, compared to ultrasounds. They can propagate with a little loss of energy and the tissue lesions produced are minimal. Dornier Laboratories in Germany have been the first to study the use of the shock waves in the urolithiasis treatment. Hoff, Behrend and Hausler have demonstrated this action against the rocks. Because of the multiple experiences regarding the surgically removed calculi, then on animals, on February 7, 1980, the first patient suffering from urinary lithiasis was treated with ESWL at the Urology Clinic of the University of Medicine in Munchen. Also in 1980, the first clinical study on a lot of 21 patients, treated by extracorporeal lithotripsy, was published [**12,13**]. The studies have continued, many centers of extracorporeal lithotripsy have appeared, and new generations of lithotripters have been proposed and developed, revolutionizing the treatment of the urinary lithiasis. This new technique has radically changed the therapeutic tactics of the urinary system calculi. The experience has proved that ESWL is a certain and efficient method, being appreciated in the whole world as one of the main methods of treatment in urolithiasis [**3,15,16**].

Starting with 1990, the extracorporeal lithotripsy has been implemented in the Republic of Moldova, in the Urology and Nephrology Clinic of the Clinical Republican Hospital [**6**], first by using the electrohydraulic method of disintegration, and then by modernizing the methods, a device with an electromagnetic principle of disintegration of the calculi has been bought in 1996. This method has radically changed the concepts and the strategy of the urology specialists regarding the treatment of urinary lithiasis, decreasing the number of the surgeries, the mortality rate and the hospitalization period of the patients [**10**]. The efficiency of the method varies according to the latest data in 80-90% of the cases [**12,13,18**]. Other authors describe the results as reaching 95-98%, depending on the presence of the factors that contribute to the success of applying the given method [**23,24**]. Simultaneously with the successes of ESWL, there are also failures, having a frequency of 18-25% [**19,29**]. For the improvement of the ESWL results in the treatment of the urinary lithiasis, the following factors should be taken into account: the localization of the calculus, its dimensions, the period of its persistence, the presence of the urinary infection and the kidney inflammatory process, the structure and its chemical composition, the density of the calculus, etc. [**13**]. The use of the method, its indications and contraindications are in the permanent attention of the specialists, without any unanimous opinions. In 2003, Yamauchi T [**33**] believed that the description of the relative and absolute indications and the contraindications for the extracorporeal lithotripsy are vague and empirical. Sinescu I [**30**] notes that the indications of the treatment regarding the dimensions of the calculi, starting with the 1980s until present, have undergone important changes, due to the implementation of the new generation lithotripters in the medical practice. It has been shown that the efficiency of ESWL depends on the model and type of the lithotripter. The small size calculi are better dezintegrated by the piezoelectric lithotripters (“Piezolith”, “EDAP”, “Litoring”) and the big size ones by the electromagnetic lithotripters (“Litostar”, “Dornier”, “Modulit”, “YpaT”). In 2002, Simion and al. stated that the ESWL treatment of the R-g negative calculi is difficult due to the impairment in the visibility of the calculus [**29**]. In 2001, Tiselius and al. [**31**] proposed in their works, the administration of contrast substances, intravenously, in the treatment of the R-g negative calculi with ESWL. These are compounds of uric acid, their focusing this way increasing the efficiency of the method and avoiding the ureteral catheterization. In 2004, Putman and al. [**26**] reported that, usually, the radiolucent calculi can cause troubles and multiple complications. In these cases, the authors give priority to other methods. In order to visualize the R-g negative calculi and avoid the intravenous injection of contrast substances, an alternative method has been proposed: the use of shunt or the retrograde urethrography [**26,27**]. Parallel with the main factors that contribute to the efficiency of the extracorporeal lithotripsy, there is a series of factors that worth being taken into account, because they have their share in the results of the treatment. One of them is the presence of the urinary infection. Most authors believe that the infection is a contraindication of the treatment [**6,7**], others on the contrary, prefer to use it as antibiotics therapy, thus obtaining satisfying results [**16,17**]. There are many opinions regarding the application of treatments in these cases, the prophylactic one implies the ureteral probe, the shunt or the percutaneous nephrostomy installation (NLP) [**4**]. These manipulations are regarded as auxiliary measures by some authors, and their rate differs in literature [**13**]. Sinescu I, also supported by other sources [**25**] believes that the main condition in the application of ESWL is the presence of a negative urinalysis in patients until the treatment is undergone. In these conditions, the therapy with antibiotics is not necessary, even if with a prophylactic purpose. With the accumulation of experience and the technical achievements, with the help or not of the less invasive auxiliary procedures, it is possible, that in most of the cases, the calculi will be solved without a general or local anesthetics and a minimal rate of complications and side effects [**11,14,32**]. However, in 2006, Skolarikos and al. stated that a detailed comparison of the indications and contraindications is very complicated because the data presented in literature are very various [**28**]. The indications and contraindications of the extracorporeal lithotripsy treatment have been continuously perfected and discussed about until present; this being the reason why our study offers explanations to some of the elements that were mentioned in the work. From the appearance of the first lithotripters (Dormier HM 1 şi HM 3), they have got smaller, cheaper and more adaptable. The new generation lithotripters have double localization and guidance systems (ultrasound and fluoroscopic), allowing the specialist to compensate for the limits of the two methods of guidance used separately [**1,5,7**]. Fluoroscopy has the great advantage of being able to identify both the renal calculi and the radiopaque ureteral ones. Moreover, it can use the contrast substance to anatomically delimitate the collector system. The ultrasound shows both the radiopaque and radiolucent renal calculi, but the localization of the ureteral lithiasis is very difficult, so many times impossible [**1,2,6**].

## Aim

The aim of the study is to evaluate the efficiency, complications and limits of the shock waves of extracorporeal lithotripsy in the treatment of the reno-ureteral lithiasis, in the experience of the Urology Clinic of the Republican Clinical Hospital of applying two devices from different generations and analyzing the results obtained.

## Materials and methods

**Fig 1:**
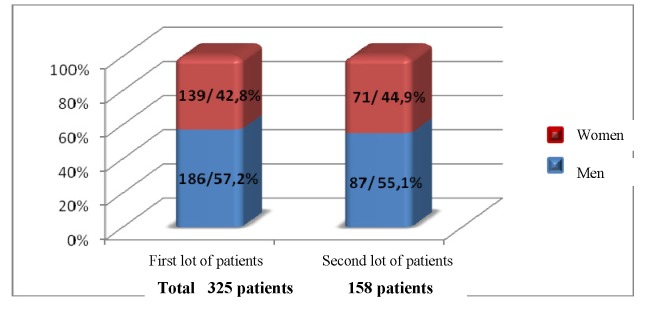
The distribution of patients according to sex (n/%)

The study was made in the Urology and Nephrology Clinic of the Republican Clinical Hospital, during January 2005 and August 2011, on a group of 484 patients diagnosed with reno-ureteral lithiasis and treated with ESWL. The patients have been distributed in two lots according to the criteria of the study. The first lot of study contained 325 patients, who have been treated by using Lithostar Multiline device, produced by “Siemens” (Germany), year of fabrication 1996, during January 2005 and April 2006 (for 14 months). The second lot consisted of 158 patients, treated with the new Modulith SLK Storz Medical lithotripter (Germany), year of fabrication 2010, equipped with a double localization and fluoroscopic and ultrasound guidance, during May-August 2011 (for 3 months). The distribution of the patients in examined lots, according to sex, is presented in**Fig. 1**

The protocol of investigations contained the following elements: the clinical examination, usual laboratory samples (including a urine and urinalysis sample), abdominal ultrasound, simple reno-vesical X-ray, intravenous urography and, in some cases, a CT scan or a spiral CT scan, retrograde uretheropielography.

**Fig 2:**
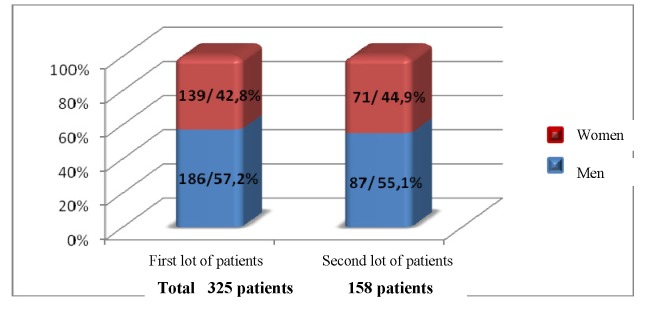
The distribution of patients according to age group

The age of the patients in the First lot has varied between 18 and 78 years, with a mean age of 45,3±12,9 years, in the Second lot the age varied between 22 and 73 years, the mean age being of 45,5±13,1 years. The distribution of the patients according to the age group is presented in **Fig. 2**.

**Fig 3:**
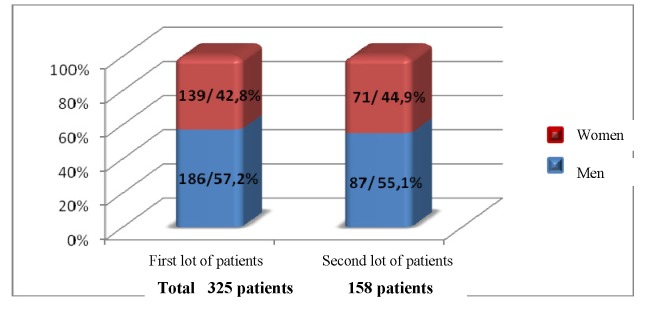
The distribution of patients according to the affected part

**Table 1:** The distribution of calculi at the renal/ ureteral level

Localization	First lot	First lot	Second lot	Second lot
	n	%	n	%
Kidney	126	38,8	63	39,9
superior calices	6	1,8	3	1,9
medium calices	1	0,3	4	2,5
inferior calices	15	4,6	5	3,2
basin	68	20,9	46	29,1
JPU	35	10,8	4	2,5
Ureter	199	61,2	95	60,1
1/3 superior	87	26,8	23	14,6
1/3 medium	17	5,2	20	12,7
1/3 inferior	96	29,5	53	33,5

In the first lot, the ESWL procedure for a single calculus was made for 321 (98,8%) patients, for multiple lithiasis – for 4 (1,2%) patients (two calculi – for 3, more than two calculi – for 1 patient). In the second lot, the extracorporeal lithotripsy for only one calculus was made for 157 (99,4%) patients, for multiple lithiasis – for 1 (0,6%) patient (two calculi). 

In the case of the patients with multiple urolithiasis, the localization of the calculi varied (pielic and caliceal renal calculi, or only caliceal, ureteral and multiple ureteral). 

**Table 2:** The distribution of calculi at the renal/ ureteral level

	First lot	First lot	Second lot	Second lot
	n	%	n	%
Radiolucent calculi	0	0	18	11,4
Radiopaque calculi	325	100	140	88,6

As it can be observed in the first lot, all the calculi were Rg. Positive, while the second lot, which represents 11,4% of the cases in the lot, contained only Rg. Negative calculi. 

The dimension of the calculi has varied between 0,3 and 18 mm (218 cases under 10 mm, 107 – between 10 and 18 mm), with the average of 0,79±0,3 cm in the first lot. In the second lot, the dimension of the calculi has varied between 0,5 and 22 mm (56 cases under 10 mm, 10 and 22 mm), with an average value of 01±0,3 cm.

The painkillers used in the ESWL proceedings have consisted of the injectable administration of analgine, ketotifen or ketorolac, etc. 

All the patients have received antialgic, antispastic, anti-inflammatory and antibacterial medication after ESWL proceedings. 

The statistics processing of the obtained results was made in MS Excel 2003 and SPSS 17 (StatSoft) and descriptive and percentage statistics have been used, the comparative analysis using the “Student” criteria. The descriptive data are presented in M±SD format, the real calculated statistic difference is at the p<0,05 level.

## Results

The criteria of results analysis were the following: rate of success, number of failures, time of eliminating the calculi, the complications and their way of solving. The “stone-free” (fragmenting and completely eliminating the calculus) rate of success is directly connected with the used lithotripter. 

In order to disintegrate the calculi in 325 patients in the first lot, 404 ESWL sessions were done, in 61 (18,8%) cases the repeating of the procedure was necessary, in 9 (2,8%) cases, the lithotripsy was needed in the third session. In the second lot, for the treatment of 158 patients, 193 ESWL sessions were done, in 33 (20,9%) cases, the ESWL was done twice and only in 1(0,6%) patient the ESWL was done thrice. 

**Table 3:** The distribution of the patients according to the stationary/ ambulator

	First lot	First lot	Second lot	Second lot
	n	%	n	%
Ambulatory	94	28,92	97	61,4
Stationary	231	71,08	61	38,6

From the data presented in **Table 3**, the raised double number of patients treated with ESWL, ambulatory, is presented, (p<0,001) in the second lot, compared to the first lot. The analgesia before the intervention was necessary in the first lot of 404 ESWL sessions in 312 (77,2%) cases, while, in the second lot, out of 193 sessions, the ESWL was necessary only in 35 cases (18,1%), having the real static difference of p<0,001.

The medium time of each lithotripsy session was between 30 and 50 minutes. The amount of impulses given to the first lot was between 4000 and 5000 (43,8% and 56,2% respectively), in the second lot the amount of impulses has varied between 3000 and 5000, predominantly of 4000 shock waves in 94,8% of the cases, in one session. 

The dimension of the calculi has varied between 0,3 and 18 mm (218 cases under 10 mm, 107 (32,9%) – between 10 and 18 mm), with a medium value of 0,79±0,3 cm in the first lot. In the second lot, the dimension of the calculi varied between 0,5 and 22 mm (56 cases under 10 mm, 102 (64,5%) – between 10 and 20 mm, and bigger than 22 mm), with the medium value of 1,01±0,3 cm. The data processing shows a significant statistical difference of (р<0,001), in favor of the second lot of study. The application of the new model of lithotripter, Modulith SLK Storz Medical, has given the possibility to perform calculi lithotripsy sessions with bigger diameters, predominantly the ones bigger than 1 cm. Approximately twice, compared to the ones in the first lot of study, it has been proved significant from the statistical point of view. 

The localization of the calculi from the ultrasound point of view was possible in the second lot for the Rg-negative calculi, which was done in 18 (11,4%) patients, which was not possible in the first lot. 

The control of the disintegration dynamics of the calculi and their centering during the sessions, with the application of fluoroscopy, attests a higher rate of application in the patients of the first lot of study, statistically demonstrated as being of (p<0,001) 5’47” ±1’28” comparatively with the values of the second lot 5’02” ±1’20.”

The disintegration and the total elimination of the calculi has been made as it follows: the first lot – the fragmenting took place in 309 (95,1 %) cases, and the rate of elimination of the fragments was of 93,5% (304 cases). In the second lot, the fragmenting of 155 (98,1%) cases, with a “stone-free” rate of 97,4%, is significantly real comparatively with the first lot of (p<0,05). 

The risks of this method are due to the possibility of complications appearance (**Table 4**), which are proportional with the intensity of the shock waves, the number of the lithotripsy sessions, the structure and the dimensions of the calculi. The evaluation of the complications of both study lots and their solving are presented in **Table 4**. 

In order to minimize the “steinstrasse” rate in the second lot, minimal endourological methods were adopted, an autostatic “JJ” urethral shunt was installed before the ESWL procedure in 15 (9,5%) cases and post lithotripsy in 7 (4,4%) cases. These have significantly diminished the appearance of complications. The failure of the treatment occurred in 6,2% (20 cases) in the first lot and 3,2% (5 cases) in the second lot of study. The solving of these cases was done by ureteroscopy and open surgical treatment. One death was registered in the first lot, being caused by a post ESWL thromboembolism.

**Table 4:** The major complications post-ESWL and their solving

Complication	First lot	First lot	First lot	Second lot	Second lot	Second lot	p
	Nr	%	solving	Nr	%	solving	
Acute pyelonephritis	12	3,7	Ureteral Catheterization Antibiotics	1	0,6	Ureteral Catheterization Antibiotics	<0.05
Subcapsular Hematoma	1	0,3	Open drainage	0	-	-	-
„Steinstrasse”	30	9,2	Analgesic Spasmolytics Ureteral Catheterization	12	7,6	Analgesic Spasmolytics Ureteral Catheterization	>0.05
Death	1	0,3	-	0	-	-	-

## Discussions

The treatment of the renal lithiasis implies the appeal to modern means, such as the ESWL type, ureteroscopy or percutaneous nephrolithotomy; classical pielolitotomy was practiced in the selected cases. The shock waves extracorporeal lithotripsy imposed in the whole world as a method of first intention for the treatment of the urinary calculi, being the less invasive of all the methods (it does not lack complications), which covers 80-90% of the indications of treatment [**1,6,7**]. 

The European Guidelines on Urolithiasis recommends the active treatment with ESWL in all the calculi with sizes between 6 and 7 mm [**13**].

The factors that influence the success of the extracorporeal lithotripsy are the following: the size of the calculus, its localization, the chemical composition, the multiplicity of the calculi, the anatomical particularities. The calculi that are over 15 mm need more sessions in order to be fragmented, like the monohydrate oxalate calcium calculi. The uric acid calculi, the dehydrated oxalate calcium calculi and the phosphate-ammoniac-magnesium calculi have proved to be easier to fragment. The ESWL results are worse in the approach of the inferior caliceal calculi, the “stone-free” rate being of 41-70% [**1,8,9,21**].

There are still many controversies regarding the efficiency of different models of lithotripters [**8**], however, the Modulith SLK Storz Medical (Germany) lithotripter which is used in our clinic has proved to be very efficient. 

In order to do the surgery in optimal conditions, the collaboration with the patient is necessary, especially in the cases in which the surgery without analgesia was chosen. This is also very important after the surgery, especially the way the patient understands how he should follow the urologist’s indications (the diuresis diet, respecting the prescribed medication, periodic checkup). No major complications were highlighted in the studied lots, although they were theoretically possible, the most powerful of all being the perirenal hematoma or the urosepsis. As it was mentioned before, the “steinstrasse” was solved favorably in most of the cases. In our experience, the post ESWL hematuria is considered normal, being rarely present for more than 24 hours and less significant form the point of view of the intensity. 

## Conclusions

1. The modern lithotripters and the use of new, performing technologies offer the possibility of enlarging the sizes’ range, the chemical structure of the calculi and the ESWL list of indications. 

2. At present, the ESWL represents the first choice of treatment for most of the renal and uretral calculi, which are less than 20 mm. 

3. The choice of the correct indications is associated with the preventive endourological maneuvers that lead to a lower rate of complications

## References

[1] Agarwal MM, Naja V, Singh SK (2009). Is there an adjunctive role of tamsulosin to extracorporeal shockwave lithotripsy for upper ureteric stones: results of an open label randomized nonplacebo controlled study.. Urology.

[2] Albala DM, Assimos  DG, Dayman  RV (2001). Lower pole I: a prospective randomized trial of extracorporeal shock wave lithotripsy and percutaneous nephrolithotomy for lower pole nephrolithisis: initial results. J Urol.

[3] Argyropoulus  AN, Tolley  DA (2007). Optimizing Shock Wave Lithotripsy in the 21st Century. European Urology.

[4] Boja  R (2000). Chirurgia percutanată reno-ureterală.

[5] Eisenmenger  W (2001). The mechanisms of stone fragmentation in ESWL. Ultrasound in Medicine and Biology.

[6] Ceban E (2003). Tratamentul diferenţiat al calculilor ureterali.

[7] Geavlete  P (2007). Optimizing shock wave lithotripsy in the 21st century: Editorial Comment. Eur. Urol.

[8] Gettman  MT, Segura  JW (2005). Management of ureteric stones: issues and controversies. BJU lnt.

[9] Ghoneim IA (2005). Predictive factors of lower calyceal stone clearance after Extracorporeal Shockwave Lithotripsy (ESWL): a focus on the infundibu-lopelvic anatomy. Eur Urol.

[10] Goktas  S, Peskircioglu  L, Tahmay  L (2000). Is there significance of the choice of prone versus supine position in the treatment of proximal ureter stones with extracorporeal shock wave lithotripsy?. Eur. Urol.

[11] Golea  O, Oşan  V, Simion  C (2002). Ureteroscopia retrogradă rigidă în terapia calculilor ureterului terminal, post-ESWL eşuat/complicat. Rev Rom Urol.

[12] Grasso  M, Hsu J, Spaliviero  M (2008). Extracorporeal Shockwave Lithotripsy. emedicine by WebMD.

[13] (2011). Guidelines on Urolithiasis 2011.

[14] Jermini FR, Danuser  H, Mattei  A (2002). Noninvasive Anesthesia, Analgesia And Radiation-Free Extracorporeal Shock Wave Lithotripsy For Stones In The Most Distal Ureter: Experience With 165 Patients. Journal of Urology.

[15] Lindquist  K, Homlberg  G, Peeker  R (2006). Extracorporeal shock-wave lithotripsy or ureteroscopy as primary treatment for ureteric stones: a retrospective study comparing two different treatment strategies. Scand J Urol Nephrol.

[16] Lingemann JE, Lifshiz  DA, Evan  AP (2002). Extracorporeal Shock-Wave Lithotripsy. Campbell's Urology.

[17] Lingemann JE, Lifshiz  DA, Evan  AP (2003). Surgical management of urinary lithiasis, in Walsh P, Retik A, Vaughan D, Wein A. Campbell’s Urology 8th edition.

[18] Manu  R (1998). Litotripsia extracorporeală cu unde de şoc (ESWL). Urologie Clinică.

[19] McAteer JA, Bailey  MR, Williams Jr JC (2005). Strategies for improved shock wave lithotripsy. Minerva Urol Nephrol.

[20] Osan  V, Simion  C, Golea  O (2002). Eficienta ESWL pentru calculii din ureterul inferior. Rev. Rom. Urol.

[21] Pearle  MS, Lingeman  JE, Leveillee  R (2005). Prospective, randomized trial comparing shock wave lithotripsy and ureteroscopy for lower pole caliceal calculi 1 cm or less. J Urol.

[22] Pearle  MS, Nadler  R, Bercowsky  E (2001). Prospective randomized trial comparing shock wave lithotripsy and ureteroscopy for management of distal ureteral calculi. J Urol.

[23] Preminger  GM, Tiselius  HG, Assimos  DG (2007). EAU/AUA Nephrolithiasis Guideline Panel. Guidelines on urolithiasis. J Urol.

[24] Poulakis  V, Dahm  P, Witzsch  U (2003). Prediction of lower pole stone clearance after shock wave lithotripsy using an artificial neural network. J Urol.

[25] Preminger  GM (2006). Management of lower pole renal calculi: shock wave lithotripsy versus percutaneous nephrolithotomy versus flexible ureteroscopy. Urol Res.

[26] Putman  SS, Hamilton  BD, Johnson  DB (2004). The use of shock wave lithotripsy for renal calculi. Curr Opin Urol.

[27] Rassweiller  JJ, Renner  C, Chaussy  C (2001). Treatment of renal stones by extracorporeal shockwave lithotripsy: an update. Eur Urol.

[28] Skolarikos A, Alivazatos  G, de la Rossette J (2006). Extracorporeal shock wave lithotripsy 25 years later: complications and their prevention. Eur Urol.

[29] Simion  C, Oşan  V (2002). ESWL la calculii din ureterul lombar. Reuşită, eşec, posibilităţi de rezolvare. Revista Româna de Urologie.

[30] Sinescu  I, Gluck  G (2008). Tratat de Urologie.

[31] Tiselius  HG, Ackermann  D, Alken  P (2001). Working Party on Lithiasis, European Association of Urology. Guidelines on urolithiasis. Eur Urol.

[32] Unsal  A, Cimentepe  E, Bozoklu  A (2001). Comparative study of etofenamate and fentanyl for outpatient extracorporeal Shockwave lithotripsy. Scand J Urol Nephrol.

[33] Yamauchi  T, Tsukamoto  T, Mori  Y (2003). Ureteral stricture after ESWL for ureteral calculi. Nippon Hinyokika Gakkai Zasshi.

